# The surgical plane for lingual tonsillectomy: an anatomic study

**DOI:** 10.1186/s40463-016-0137-3

**Published:** 2016-04-05

**Authors:** Eugene L. Son, Michael P. Underbrink, Suimin Qiu, Vicente A. Resto

**Affiliations:** Department of Otolaryngology - Head and Neck Surgery, University of Texas Medical Branch, 301 University Boulevard, Galveston, TX 77555 USA; Department of Pathology, University of Texas Medical Branch, 301 University Boulevard, Galveston, TX 77555 USA

**Keywords:** Lingual tonsil, Surgical plane, Transoral robotic surgery, Lingual tonsillectomy

## Abstract

**Background:**

The presence of a plane between the lingual tonsils and the underlying soft tissue has not been confirmed. The objective of this study is to ascertain the presence and the characteristics about this plane for surgical use.

**Methods:**

Five cadaver heads were obtained for dissection of the lingual tonsils. Six permanent sections of previous tongue base biopsies were reviewed. Robot assisted lingual tonsillectomy was performed using the dissection technique from the cadaver dissection.

**Results:**

In each of the 5 cadavers, an avascular plane was revealed deep to the lingual tonsils. Microscopic review of the tongue base biopsies revealed a clear demarcation between the lingual tonsils and the underlying minor salivary glands and muscle tissue. This area was relatively avascular. Using the technique described above, a lingual tonsillectomy using TORS was performed with similar findings from the cadaver dissections.

**Conclusions:**

A surgical plane for lingual tonsillectomy exists and may prove to have a role with lingual tonsillectomy with TORS.

## Background

The base of tongue had once been a difficult area for surgery to perform on because of problems with exposure. With new innovations in endoscope technology, transoral laser microsurgery and transoral robotic surgery (TORS) with the da Vinci Surgical System manufactured by Intuitive Surgical, Inc., access to the tongue base has been made more feasible. In the base of tongue, the lingual tonsils are an important target for surgery. There are two main indications for lingual tonsillectomy.

The first indication is lingual tonsil hypertrophy (LTH), which can contribute to obstructive sleep apnea (OSA) in pediatric and adult patients. LTH among other functionally or fixed areas of obstruction in the upper aerodigestive tract are targets for sleep surgery. In pediatric patients, LTH can be primary or a secondary following a tonsillectomy and adenoidectomy [1].

The second indication is for squamous cell carcinoma of unknown primary (SCCUP) in the head and neck [2]. There has been an increase in the incidence of human papilloma virus (HPV) related oropharyngeal squamous cell carcinoma [3]. A large of number of SCCUP with negative clinical and radiographical evidence of a primary tumor are most commonly found to have primaries in the palatine and lingual tonsils. More recently, performing palatine tonsillectomy and lingual tonsillectomy has returned finding the primary tumor in 75–90 % allowing patients to receive a decreased amount of radiation lessening side effects [3, 4].

Lingual tonsillectomy techniques include cold dissection, electrocautery, coblation, carbon dioxide (CO_2_) laser and microdebrider [5–9]. Many of these methods ablate or disrupt the microarchitecture of the lingual tonsils. In SCCUP, carpet resection of the lingual tonsils requires the desired tissue to be left intact for diagnosis under a microscope by a pathologist. Lingual tonsillectomy techniques have been described but no evidence of a surgical plane of dissection is available. Some have described the presence of a potential capsule and some have stated there is none. Joseph et al. described a layer of fibrous tissue that sometimes delineates this tissue from the tongue but no definite capsule [10]. Multiple authors in the early 20th century described a potential plane as “a basement membrane analogous to the capsule of the faucial tonsils” but not as delineated or developed as that tissue [11–14]. Dundar et al. describes the lingual tonsil having no capsule [5]. Lin and Koltai in their case series of coblation of lingual tonsillectomy in 26 pediatric patients describe no clear demarcation between the lingual tonsils and the tongue musculature, although the change in tissue quantities becomes readily apparent [1]. We believe that deep to the lingual tonsils, a relatively avascular plane made up of connective tissue exists. This potential surgical plane may be utilized in lingual tonsillectomy for the aforementioned indications.

## Methods

This study was approved by the institutional review board from the University of Texas Medical Branch. Five fresh cadaveric heads were procured for the purpose of this study. These cadavers were ages ranged from 86 years-old to 101 years-old including 2 females and 3 males.

### Cadaver dissection

The tongue from 5 cadaver heads were removed. Five complete dissections of the lingual tonsils were performed with scissors and forceps. First, a midline incision is made from the foramen cecum to the vallecula. An incision is also made immediately posterior to the circumvallate papilla. The anterior medial edge of a side of the lingual tonsils were grasped with forceps and then, dissection with the Iris scissors was performed in the lateral posterior direction until the lingual tonsils were removed en bloc on each side. Grossly, the muscle was identified as darker red in color and striations present. The dissection is carried to the border of the lateral pharyngeal wall and posteriorly to the edge of the vallecula. Care was take to remove the lymphoid tissue while keeping the underlying muscle intact. Digital photography was performed during the dissections.

### Histological sectioning

Six archived permanent sections of base of tongue biopsy specimens from the department of pathology at University of Texas Medical Branch were reviewed. Three cases with no malignancy detected and three cases with dysplasia were chosen for recording of digital photography under magnification. These specimens were from base of tongue biopsies from patients with SCCUP and had been fixed in formalin, processed for paraffin embedding and stained with hematoxylin and eosin. These specimens were reviewed for histological characterization of the lingual tonsils.

### Robot-assisted lingual tonsillectomy

A 52 year old male with SCCUP in the left neck underwent robot assisted lingual tonsillectomy as well as bilateral palatine tonsillectomy. A Fehy-Kastenbauer mouth gag was used for access to the tongue base. The da Vinci Surgical System was brought into the surgical field with visualization of the lingual tonsils. The 30-degree angled endoscope was placed in the midline, and the two working robotic arms were placed in the appropriate position. A Maryland dissector was used on the right arm and a monopolar cautery hook was used on the left arm for the left sided lingual tonsillectomy. The robot was then used to assist taking the lingual tonsil tissue down to the muscle layer, starting medially and dissecting laterally. A midline incision is made from the foramen cecum to anterior to the median glossoepiglottic fold. An incision is also made immediately posterior to the circumvallate papilla. The anterior medial edge of a side of the lingual tonsils were grasped and dissection was performed in the lateral posterior direction until the lingual tonsils were removed as one specimen for each side. Care was take to remove the lymphoid tissue keeping the underlying musculature intact. The specimens were then sent to the pathology department for histological review.

## Results and discussion

### Cadaver dissection

**Fig. 1:**
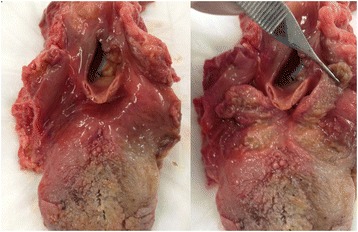
Gross dissection of lingual tonsils is shown. Left image shows the lingual tonsils before dissection and the right image shows the lingual tonsils dissected and reflected posteriorly

In all five cadaver dissections, the plane between the lingual tonsils and the underlying musculature was identified and used for excision of the lingual tonsils. There was a plane of dissection easily separating the lingual tonsils and the underlying tongue musculature. No grossly visible vessels nor nerves were encountered during the dissections (Fig. 1).

### Histological sectioning

**Fig. 2:**
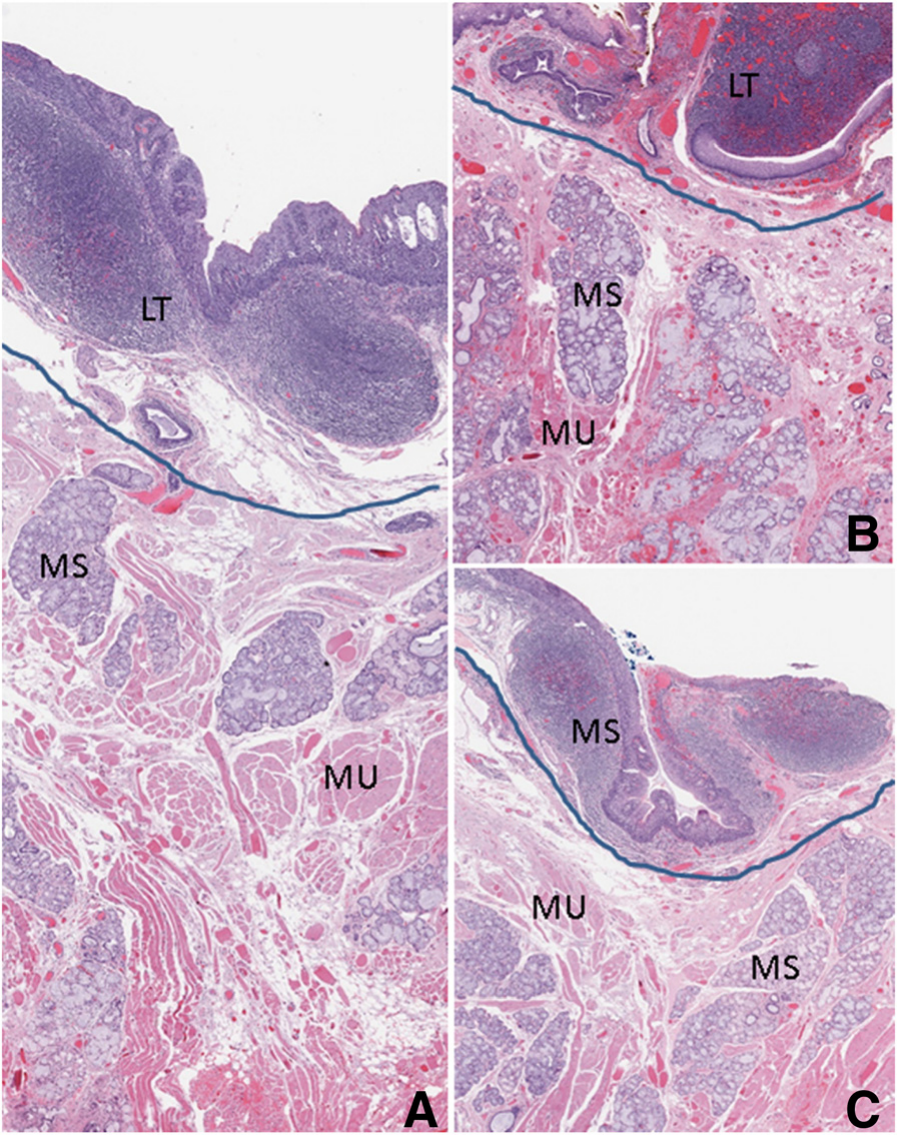
Permanent sections of base of tongue biopsies with benign pathology. Blue line demarcates surgical plane. Lingual tonsils (LT), minor salivary gland tissue (MS), muscle (MU) are labeled. Presence of submucosal edema exageratign plane in **a** compared to no edema in **b** (hypervascular) and **c**

**Fig. 3:**
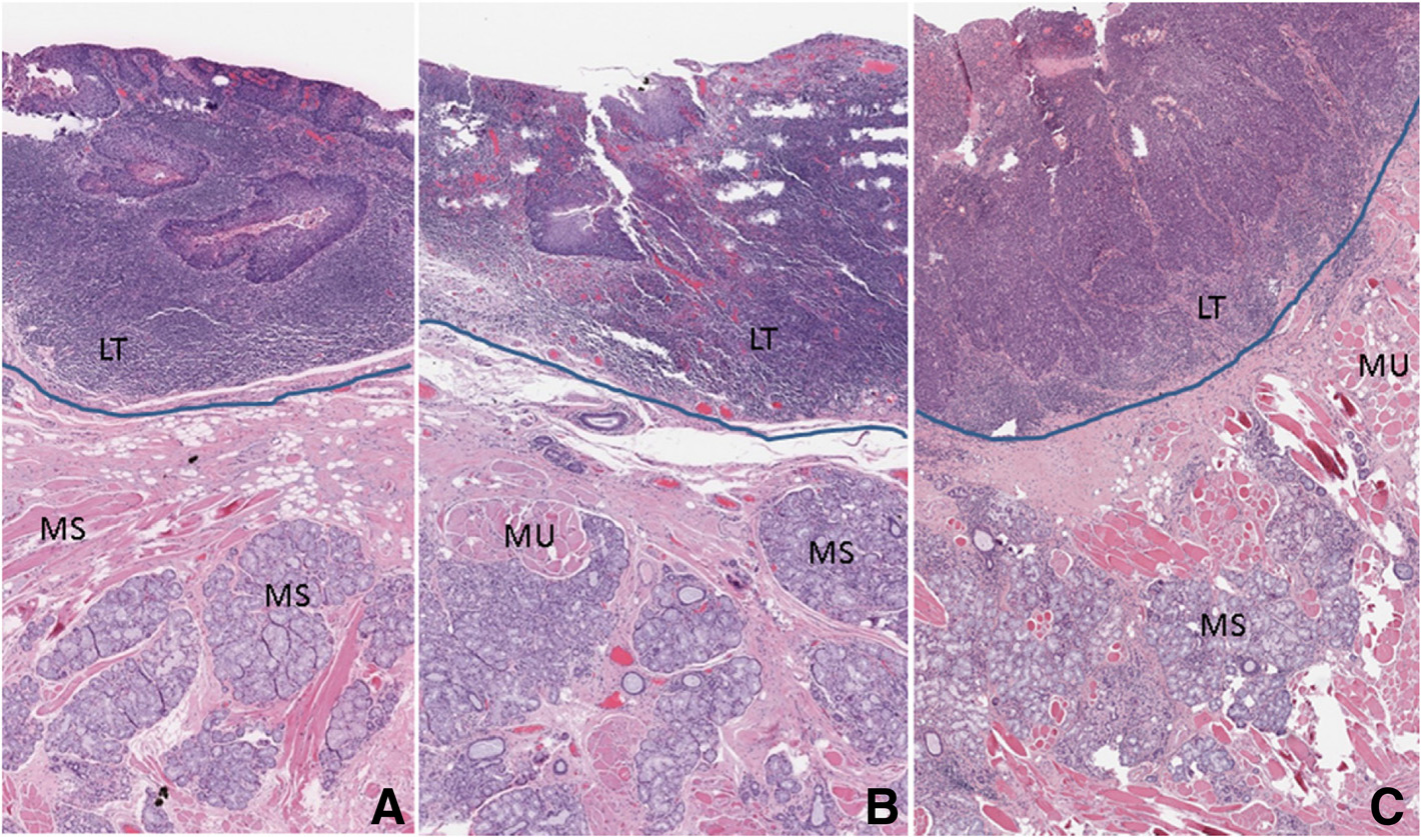
Permanent sections of base of tongue biopsies with premalignant pathology. Blue line demarcates surgical plane. Lingual tonsils (LT), minor salivary gland tissue (MS), muscle (MU) are labeled. Plane ispreserved in the presence of dysplasia in **a** and **b** (most defined plane). However, peritumoral lymphocytes infiltrate obscure the space between the lingual tonsil and the underlying muscle in **c**

In these biopsies of the lingual tonsils, there was a relatively less vascular or avascular area between the lingual tonsil and the underlying minor salivary glands and muscle tissue in all cases (Figs. 2 and 3). Between the lingual tonsil and the submucosal muscular tissue at the base of the tongue a distinct space or line is demonstrated in both benign (Fig. 2) and premalignant (Fig. 3) cases. The space indicated by the drawn line is less vascular or avascular, by which the squamous mucosa with the lingual tonsil is distinct from the minor salivary gland and the muscle. The minor salivary gland and muscle is intimately admixed especially in the superficial portion of the muscle. Presence of submucosal edema (Fig. 2a) may exaggerate the space and presence of dysplasia associated with peritumoral lymphocytes infiltrate may obscure the space between the lingual tonsil and the underlying muscle as showed in Fig. 3c. However, in all conditions, dissection between two layers are feasible based on histological study.

### Robot-assisted lingual tonsillectomy

**Fig. 4:**
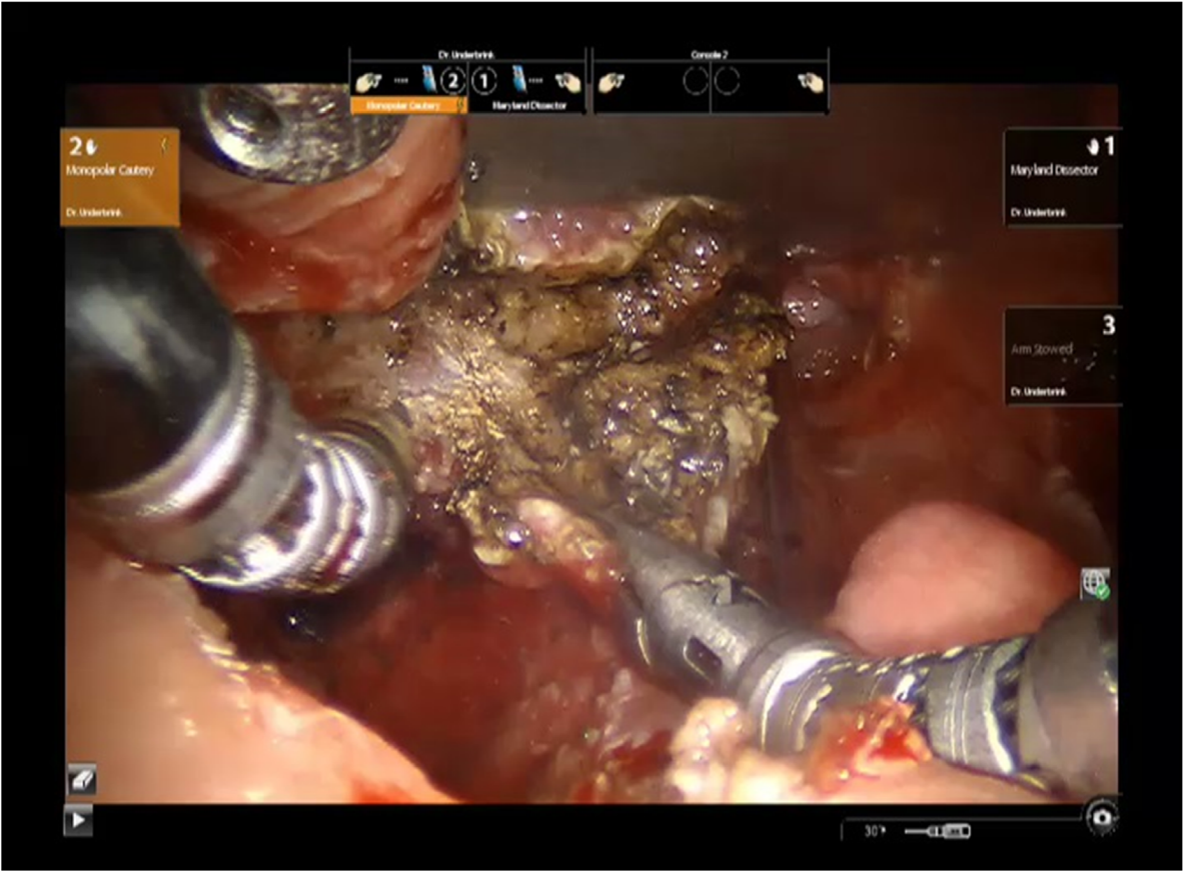
Intra-operative photography of lingual tonsillectomy during TORS. Lingual tonsil is grasped and reflected posteriorly

The procedure was performed uneventfully. No grossly visible vessels nor nerves were encountered during the dissections (Fig. 4). The patient had a normal post-operative course without any complications. Microscopic examination of this specimen showed microscopic foci of squamous cell carcinoma for which a random biopsy of the base of tongue would have missed.

The palatine tonsils have a well-described plan separating it from the surrounding oropharyngeal musculature. There has been only speculation about the existence of a surgical plane for the lingual tonsils, being only described anecdotally in the current literature. In our study, we show a relatively avascular plane deep to the lingual tonsils in cadaver dissection, in histologic sections and in vivo.

In the five dissections performed, there was a space deep to the tonsils that gave less resistance in the force of dissection. In Fig. 1, there a is an uneven layer deep to this plane after dissection representing the minor salivary glands associated with the muscle layer as seen in the histological sections. In this plane, there were no grossly visible neurovascular structures appreciated for all specimens. There may be a feasible way for cold dissection of the tonsils using direct visualization or with robot assistance with a potential for decreased amount of cautery, minimal bleeding and decreased post-operative morbidity. This plane may also be utilized instead of ablative techniques such as in obstructive sleep apnea in pediatric patients to prevent secondary hypertrophy of the lingual tonsillar after tonsillectomy and adenoidectomy for obstructive sleep apnea.

Decreased use of cautery for dissection and hemostasis may provide better tissue diagnosis when lingual tonsillectomy is performed for elucidation of a diagnosis of an unknown head and neck primary tumor. In the case described, the diagnosis was made using a carpet resection of the lingual tonsils using our technique. Random biopsies of the base of tongue may miss a diagnosis making the consequential treatment different.

## Conclusions

The lingual tonsils have become more accessible in recent times for surgical intervention for diagnostic and treatment purposes. There is an avascular plane for dissection deep to the lingual tonsils and superficial to the underlying minor salivary glands and lingual musculature. This plane may be utilized in surgical resection in appropriate patients to potentially decrease post-operative morbidity and increase diagnostic yield. Further studies in human subjects with this plane utilized will need to be performed in the future.

## Abbreviations

CO_2_: carbon dioxide
HPV: human papilloma virus
LTH: lingual tonsil hypertrophy
OSA: obstructive sleep apnea
SCCUP: squamous cell carcinoma of unknown primary
TORS: transoral robotic surgery
